# Clinical characteristics, risk factors, and surgical outcomes of secondary macular hole after vitrectomy

**DOI:** 10.1038/s41598-019-55828-x

**Published:** 2019-12-20

**Authors:** Hyun Goo Kang, Jae Yong Han, Eun Young Choi, Suk Ho Byeon, Sung Soo Kim, Hyoung Jun Koh, Sung Chul Lee, Min Kim

**Affiliations:** 1 Department of Ophthalmology, Institute of Vision Research, Gangnam Severance Hospital, Yonsei University College of Medicine, 06273 Eonjuro 211, Gangnam-gu, Seoul, Republic of Korea; 2 Department of Ophthalmology, Institute of Vision Research, Severance Eye Hospital, Yonsei University College of Medicine, 03722 Yonsei-ro 50-1, Seodaemun-gu, Seoul, Republic of Korea

**Keywords:** Retinal diseases, Vision disorders

## Abstract

Secondary macular hole(MH) formation after vitrectomy is rare and its risk factors and pathogenesis are not clearly understood. This retrospective study was conducted to identify the risk factors of this complication and assess outcomes at 2 tertiary centres. The primary outcomes were the clinical characteristics associated with development of secondary MH, which included the primary diagnosis for initial vitrectomy, features on optical coherence tomography, and adjuvant surgical techniques used during the initial surgery. Secondary outcomes included the change in best-corrected visual acuity(BCVA), clinical factors associated with the need for re-operations for MH closure and prognostic factors for the visual outcomes. Thirty-eight eyes out of 6,354 cases (incidence 0.60%) developed secondary MH after undergoing vitrectomy for various vitreoretinal disorders over an 11-year period, most frequently after initial surgery for retinal detachment(RD) (9 eyes) and secondary epiretinal membrane (6 eyes). The mean age was 57.1 years (range: 17.8–76.7), and the mean follow-up was 51.1 months (range: 6.8 to 137.6). Prior to secondary MH formation, development of ERM was the most common OCT feature (19 eyes, 50%), and no cases of cystoid macular oedema (CME) were observed. A greater proportion of eyes with secondary MH had long axial lengths (32% ≥26 mm vs 5% of eyes ≤22 mm). MH closure surgery was performed in 36 eyes and closure was achieved in 34 (success rate 94%, final BCVA 20/86), with ≥3-line visual gain in 18 cases. BCVA at MH onset (OR = 0.056, P = 0.036), BCVA at post-MH surgery month 3 (OR = 52.671, P = 0.011), and axial length ≥28 mm (OR = 28.487, P = 0.030) were associated with ≥3-line visual loss; a history of macula-off RD (OR = 27.158, P = 0.025) was associated with the need for multiple surgeries for MH closure. In conclusion, secondary MH occurs rarely but most commonly after vitrectomy for RD. Patients with axial length ≥28 mm and poor BCVA at 3 months post-operation may have limited visual prognosis; those with a history of macula-off RD may require multiple surgeries for hole closure.

## Introduction

Secondary full-thickness macular hole (MH) formation after vitrectomy is a rare surgical complication, with an incidence reportedly between 0.24% and 1.9%^1–3^. Unfortunately, the factors contributing to the development of a postoperative secondary MH in the absence of intact vitreous are not well-understood, and pathogenic mechanisms other than those for idiopathic MH may be involved. An idiopathic MH is generally believed to be formed as a result of interactions between various forces on the fovea, mainly from anteroposterior and tangential traction by the vitreous^4,5^. Therapeutic surgical intervention thus attempts to relieve these tractional components through vitrectomy, with or without internal limiting membrane (ILM) peeling, with favourable outcomes^6,7^. However, for post-vitrectomized eyes, the thorough detachment of the vitreous from the posterior pole and its removal eliminates these crucial pathogenic elements.

Current literature describes cases of secondary MH after surgical repair of retinal detachments (RD)^8^, epiretinal membranes (ERM)^2^, vitreomacular traction syndrome (VMTS)^9^, and myopic foveoschisis^10^. These studies also document a wide combination of adjuvant techniques used during the initial vitrectomy, such as ILM peeling, scleral buckling, and pneumatic tamponade^11^. Many of the aforementioned studies also report that the vitreous was already detached prior to the primary vitrectomy^12^. Suggestions for possible aetiologies for secondary MH formation include foveal cystic degeneration, iatrogenic trauma, secondary ERM tangential contraction, and vitreoschisis^8^. However, the small sample sizes in these previous studies, wide diversity of clinical characteristics, and varied outcomes pose difficulties in identifying common risk factors for the development of secondary MH.

In this present study, we analysed a relatively large number of cases to investigate the clinical spectrum of a secondary MH that develops after vitrectomy and assess the treatment outcomes. Additionally, we sought to identify the risk factors that contribute to the development of secondary MH and the requirement of multiple surgeries for MH closure, and determine the predictive factors for long-term visual prognosis.

## Results

A total of 38 eyes from 37 patients were included in this study, identified from 6,354 cases of patients who underwent vitrectomy, with an incidence of 0.60%. The baseline characteristics are summarized in Table 1. Secondary MH was diagnosed after a median duration of 2.3 months after the initial primary vitrectomy. The mean age at MH onset was 57.1 years, although it ranged from 17.8 to 76.7 years, and the mean onset BCVA was 1.02 (Snellen 20/210). There was no apparent predilection for sex; however, a greater proportion of the diseased eyes had long axial lengths (5% of eyes ≤ 22 mm vs. 32% ≥26 mm). The mean total follow-up duration was 51.1 months (range: 6.8 to 137.6 months).

**Table 1:** Baseline patient characteristics (37 patients, n = 38 eyes).

Sex, no. (%)	
Male	18 (49)
Female	19 (51)
Hypertension, no. (%)	9 (24)
Diabetes mellitus, no. (%)	9 (24)
Age at primary vitrectomy, years, mean ± SD (range)	55.7 ± 14.1 (17.6–75.4)
Age at onset of MH	57.1 ± 14.6 (17.8–76.7)
Median time to MH diagnosis after vitrectomy, months (range)	2.3 (0.4–90.9)
Prior vitrectomy operations, no. (range)	1.2 ± 0.5 (1–3)
BCVA, logMAR, mean ± SD (Snellen)	
Prior to primary vitrectomy	1.19 ± 0.9 (20/313)
Onset of MH	1.02 ± 0.6 (20/210)
Axial length, mm, mean ± SD (range)	25.1 ± 2.7 (21.5–32.3)
Short eye (≤22 mm), no. (%)	2 (5)
Average eye (22–26 mm), no. (%)	24 (63)
Long eye (≥26 mm), no. (%)	12 (32)
Very long eye (≥28 mm), no. (%)	6 (16)
Pseudophakic status, no. (%)	28 (74)

BCVA = best-correct visual acuity; logMAR = logarithm of minimum angle of resolution; MH = macular hole; SD = standard deviation.

The primary diagnoses for the initial vitrectomy, co-existing ocular pathologies detected on multimodal imaging, and adjuvant techniques used can be found in Table 2. The most common primary diagnoses for the initial vitrectomy, when defined as the principal reason for initial surgical treatment, were rhegmatogenous RD (9 eyes, 24%; 5 macula-off cases) and secondary ERM (6 eyes, 16%). When combining cases of primary rhegmatogenous RD (9 eyes) with those undergoing silicone oil removal after successful repair of RD with silicone oil tamponade (oil-filled status, 5 eyes) and those having had a history of RD (n = 3), we found that altogether 17 cases (45%) had some association with RD.

**Table 2:** Diagnoses, associated ocular pathologies, treatment factors, and imaging characteristics with regard to the primary initial vitrectomy.

**Primary diagnosis, no. (%)**	38 (100)
Rhegmatogenous RD (5 macula-off)	9 (24)
Secondary epiretinal membrane	6 (16)
Vitreous haemorrhage (4 PDR, 1 PCV)	5 (13)
Oil-filled status after RD surgery (4 macula-off)	5 (13)
Idiopathic epiretinal membrane	4 (11)
Lamellar hole	3 (8)
Submacular haemorrhage (2 PCV, 1 RAM)	3 (8)
Vitreomacular traction syndrome	2 (5)
Optic disc pit-associated maculopathy	1 (3)
**Co-existing pathology and clinical features prior to initial vitrectomy, no. (%)**	
Epiretinal membrane detected on OCT	22 (58)
Vitreous attachment at fovea on OCT	22 (58)
Vitreous haemorrhage	11 (29)
PDR	4 (11)
Breakthrough from SMH	3 (8)
PCV	2 (5)
Branch retinal vein occlusion	1 (3)
RAM	1 (3)
Retinoschisis	5 (13)
Lamellar macular hole	4 (11)
Past surgical history of macula-off RD	3 (8)
Prior scleral encircling or buckling for RD	3 (8)
Proliferative vitreoretinopathy	3 (8)
Posterior uveitis	2 (5)
Prior oil removal history after RD	1 (3)
Serous neurosensory RD	1 (3)
RAM	1 (3)
**Adjuvant techniques for primary vitrectomy, no. (%)**	
ILM peeling	20 (44)
Pneumatic tamponade (SF_6_ or C_3_F_8_ gas)	16 (42)
Silicone oil injection	12 (32)
Scleral buckle/encircling	5 (13)
Intravitreal anti-VEGF injection performed	12 (32)
No. of injections, mean ± SD (range)	2.6 ± 2.0 (1–7)
Intravitreal Ozurdex® injection performed	3 (8)
No. of injections, mean ± SD (range)	1.7 ± 1.2 (1–3)

ILM = internal limiting membrane; OCT = optical coherence tomography; PDR = proliferative diabetic retinopathy; RAM = retinal arterial macroaneurysm; RD = retinal detachment; SD = standard deviation; SMH = submacular haemorrhage; VEGF = vascular endothelial growth factor.

Review of the multimodal imaging data of all involved eyes, before the initial vitrectomy, revealed a wide variety of co-existing vitreomacular pathologies in addition to the primary diagnosis, such as ERM detected in 22 eyes (58%), vitreous haemorrhage in 11 eyes (29%), retinoschisis in 5 eyes (13%), and lamellar holes in 4 eyes (11%) (Table 2). An intact subfoveal inner segment/outer segment line was observed on the OCT of 16 eyes (73%) pre-operatively and only 8 eyes (35%) post-initial vitrectomy. The mean central macular thickness was measured at 215.8 ± 173.1 μm preoperatively and 113.4 ± 61.8 μm post-operatively.

Next, we have summarized the clinical features detected after secondary MH formation (post-initial primary vitrectomy), and the intraoperative factors and surgical outcomes for MH closure in Table 3. We detected ERM in 19 eyes (50%): recurred ERM in 14 eyes and newly developed ERM in 5. We observed that all 4 cases of idiopathic ERM exhibited recurrence following the initial primary vitrectomy. No cases of cystoid macular oedema (CME) were detected prior to secondary MH formation.

**Table 3:** Clinical features after secondary macular hole formation: intraoperative factors and surgical outcomes.

MH size on pre-operative OCT, μm, mean ± SD (range)	543.9 ± 324.1
ERM on OCT (after the initial vitrectomy but prior to MH surgery), no. (%)	19 (50)
Newly formed after the initial primary vitrectomy	5 (13)
Recurrence after the initial primary vitrectomy & ERM removal	14 (37)
Surgery performed for MH closure, no. (%)	36 (95)
**Adjuvant techniques during vitrectomy, no. (%)**	
ILM peeling	29 (81)
ILM transplantation	3 (8)
No ILM staining observed	3 (8)
Silicone oil injection	7 (19)
Heavy silicone oil injection	1 (3)
**Pneumatic tamponade**	
C_3_F_8_ gas injection	26 (72)
SF_6_ gas injection	6 (17)
Autologous platelet concentrate injection	9 (25)
Closure of MH achieved, no. (%)	34 (94)
No. of operations to MH closure, mean ± SD (range)	1.3 ± 0.5 (1–3)
Multiple operations required, no. (%)	10 (28)
**Visual outcomes in BCVA, logMAR, mean** ± **SD (Snellen)**	
At post-operation 3 months (38/38 eyes)	1.00 ± 0.6 (20/199)
At post-operation 6 months (38/38 eyes)	0.86 ± 0.6 (20/144)
At post-operation 12 months (30/38 eyes)	0.78 ± 0.6 (20/119)
At post-operation 24 months (26/38 eyes)	0.63 ± 0.5 (20/86)
Overall BCVA by the most recent follow-up	0.77 ± 0.7 (20/119)
Mean BCVA change from MH onset to final visit, logMAR,, mean ± SD	−0.25 ± 0.6
Improved at least 3 lines, no. (%)	18 (47)
Decreased in 3 lines, no. (%)	7 (18)
Follow-up duration, months, mean ± SD (range)	51.1 ± 39.1 (6.8–137.6)

BCVA = best-corrected visual acuity; ERM = epiretinal membrane; ILM = internal limiting membrane; logMAR = logarithm of minimal angle of resolution; MH = macular hole; OCT = optical coherence tomography; SD = standard deviation.

**Figure 1:**
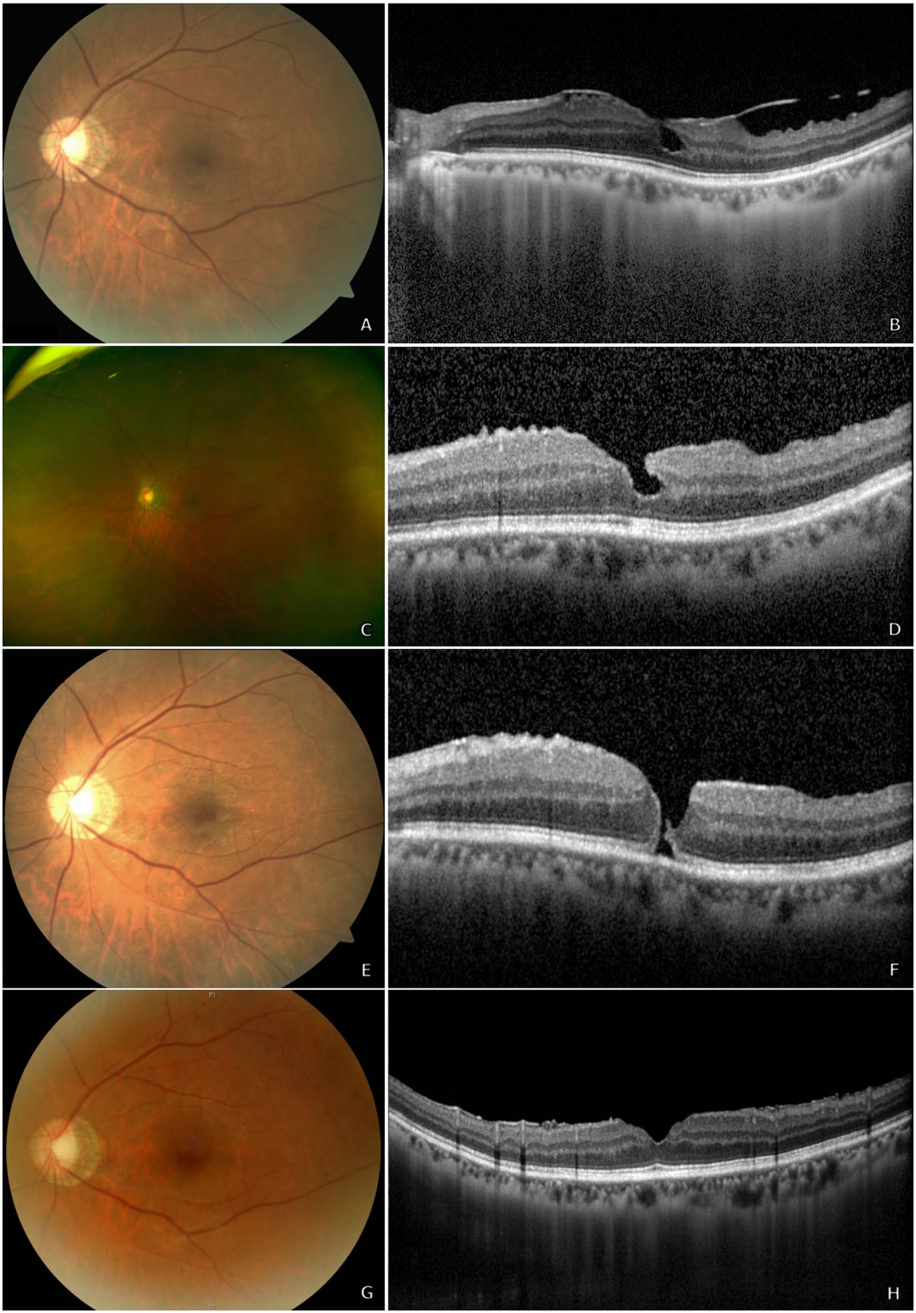
A 65-year-old woman underwent vitrectomy with internal limiting membrane (ILM) peeling for idiopathic epiretinal membrane (ERM) (**A,B**). Her best-corrected visual acuity (BCVA) was 20/133. Complete removal of ERM was noted on post-operative optical coherence tomography (OCT), although irregularity of the foveal depression remained (**C,D**). However, 1.7 months after the primary vitrectomy (BCVA 20/100), a secondary full-thickness macular hole (MH) was detected on OCT with thin ERM and no evidence of cystoid macular oedema (**E,F**). She subsequently underwent vitrectomy with ILM peeling and pneumatic tamponade with C3F8 gas. The MH remained closed 2.5 years’ post-operation, and her BCVA was 20/20 (**G,H**).

**Figure 2:**
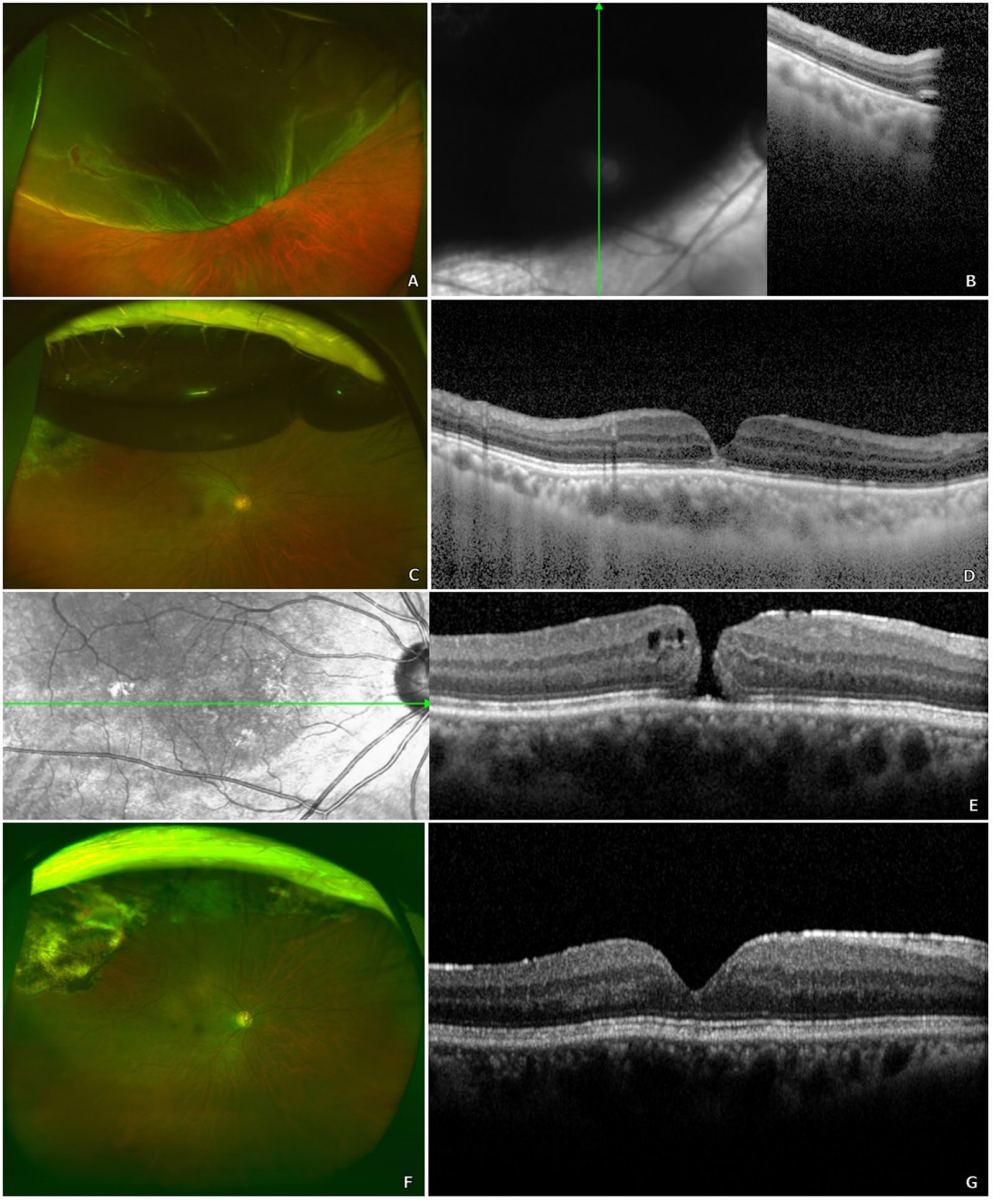
A man in his sixties underwent vitrectomy with pneumatic tamponade for macula-off retinal detachment in his right eye (**A,B**). His best-corrected visual acuity (BCVA) was 20/50. At postoperative week 5, the patient’s retina was well-attached (**C**); however, optical coherence tomography (OCT) revealed microscopic changes in foveal architecture and outer retinal layer discontinuity (**D**). Follow-up examination at postoperative week 8 (BCVA 20/67) revealed a secondary full-thickness macular hole (MH) with epiretinal membrane and without cystoid macular oedema (**E**). The patient underwent two additional vitrectomies with pneumatic tamponade and internal limiting membrane peeling for secondary MH closure. His most recent visit, at 3 years’ post-operation, revealed a BCVA of 20/29 with a closed MH, as observed on OCT (**F,G**).

**Figure 3:**
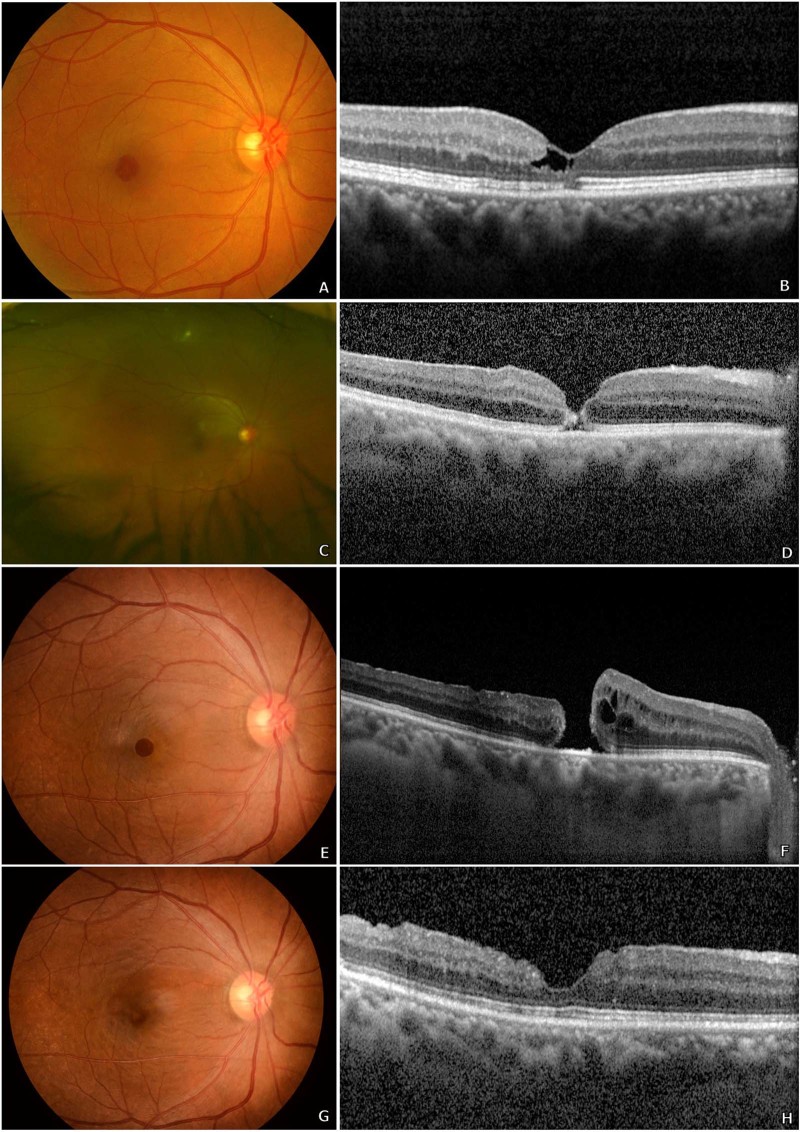
A 61-year-old woman underwent vitrectomy with wide internal limiting membrane (ILM) peeling for symptomatic lamellar hole due to type 2 macular telangiectasia (**A,B**). Her best-corrected visual acuity (BCVA) was 20/40. At postoperative day 5, imaging revealed a full-thickness macular hole (MH) with no evidence of cystoid macular oedema (**C,D**), and the patient’s BCVA decreased to 20/100. Additional pneumatic retinopexy was performed twice subsequently but the size of the MH continued to increase (**E,F**). After two further vitrectomies with ILM peeling and injection of autologous platelet concentrate, MH closure was finally achieved. Three years’ post-operation, the patient had a BCVA of 20/67 with a closed MH, as observed on optical coherence tomography (**G,H**).

**Figure 4:**
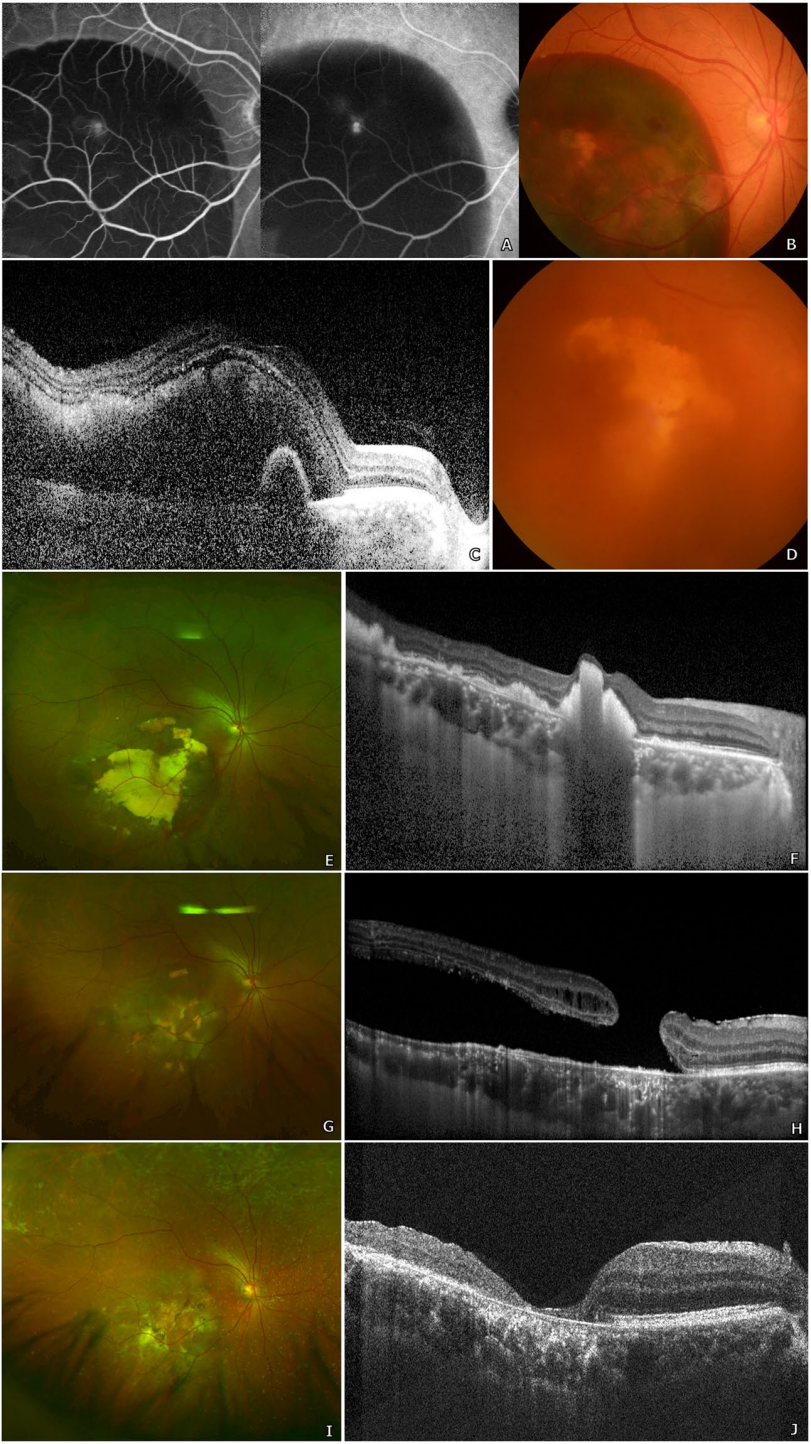
A woman in her early sixties with known submacular haemorrhage secondary to polypoidal choroidal vasculopathy, diagnosed based on typical multimodal imaging findings (**A–C**), underwent vitrectomy for breakthrough vitreous haemorrhage, which occurred after 2 intravitreal injections of anti-vascular endothelial growth factor (**D**). Her best-corrected visual acuity (BCVA) prior to initial vitrectomy was counting fingers. At post-operative month 2, multimodal imaging revealed a large subretinal hyperreflective material remaining under the fovea, although no definite macular hole (MH) was yet to be found (**E,F**). However, 2 months later, after continual intravitreal anti- vascular endothelial growth factor injections, imaging studies revealed the formation a large secondary macular hole with localized retinal detachment (**G,H**) and her onset BCVA was 20/667. She subsequently underwent multiple vitrectomies for MH closure: internal limiting membrane peeling and pneumatic tamponade was performed initially. Additional surgery with injection of autologous platelet concentrate and silicone oil was implemented. Finally, once MH closure was evidenced on optical coherence tomography (OCT), silicone oil removal operation was performed. Her most recent examination, at 2 years after initial vitrectomy, revealed a well-closed MH on OCT (**I,J**) and a BCVA of 20/200.

Surgery for MH closure was performed in 36 eyes; the intraoperative factors and treatment outcomes are summarized in Table 3. In the 2 cases that did not receive surgery, the patients refused treatment due to other debilitating illnesses, and were, therefore, managed conservatively. Successful closure, as detected on OCT, was achieved in 34 eyes (94%) after a mean 1.3 operations. In majority of the eyes, ILM peeling was attempted (32 eyes, 89%); however, no ILM staining was observed in 3 eyes (8%). Furthermore, ILM transplantation (3 eyes, 8%) and heavy silicone oil injection (1 eye, 3%) was performed in eyes requiring multiple operation for closure. Representative images from several cases can be seen in patients with idiopathic ERM (Fig. 1), macula-off RD (Fig. 2), macular telangiectasia (Fig. 3), and submacular haemorrhage (Fig. 4).

### Visual outcomes and clinical factors

The mean BCVA at the most recent follow-up was 0.77 (20/119), with the majority (24 eyes, 63.2%) having improved at least 1 line (Table 3). A visual loss of greater than 3 lines was noted in 7 eyes (18%). Multivariable analyses found that the preoperative BCVA (odds ratio [OR] = 0.056, *P* = 0.036), BCVA at postoperative month 3 (OR = 52.671, *P* = 0.030), and axial length ≥28 mm (OR = 28.487, *P* = 0.030) significantly influenced a 3-line visual loss by the final follow-up (Table 4). The size of MH was not found to be a significant factor for visual outcome in our regression analysis (OR = 1.000 (0.997–1.002), *P* = 0.726).

**Table 4:** Multivariable analyses of surgical and visual outcomes.

Factors influencing a 3-line visual gain by the most recent follow-up		
	OR (95% CI)	P value
BCVA, preoperative (prior to MH surgery) (logMAR)	9.814 (1.201–80.204)	0.033*
BCVA, post-operation 3 months (logMAR)	0.093 (0.011–0.778)	0.028*
**Factors influencing a 3-line visual loss by the most recent follow-up**		
	**OR (95% CI)**	**P value**
BCVA, preoperative (prior to MH surgery) (logMAR)	0.056 (0.004–0.825)	0.036*
BCVA, post-operation 3 months (logMAR)	52.671 (2.436–1138.719)	0.011*
Axial length ≥28 mm	28.487 (1.380–587.960)	0.030*
**Factors influencing the need for multiple surgeries for MH closure**		
	**OR (95% CI)**	**P value**
MH size on pre-operative OCT	1.004 (0.998–1.009)	0.202
BCVA, most recent follow-up (logMAR)	1.847 (0.203–16.785)	0.586
History of macula-off RD	27.158 (1.526–483.204)	0.025*
Presence of perioperative epiretinal membrane	0.231 (0.021–2.520)	0.230

BCVA = best-corrected visual acuity; CI = confidence interval; logMAR = logarithm of minimal angle of resolution; MH = macular hole; OR = odds ratio; RD = retinal detachment; *P < 0.05.

With regard to clinical factors predictive of requirement of multiple surgeries for MH closure, multivariable regression analysis revealed that a history of macula-off RD (OR = 27.158, *P* = 0.025) significantly affected the outcome of Table 4. Other factors, such as MH size on preoperative OCT (OR = 1.004, *P* = 0.202), presence of perioperative ERM (OR = 0.231, *P* = 0.230), and BCVA at the most recent follow-up (OR = 1.847, *P* = 0.586), were only significant in the univariate analyses.

## Discussion

Secondary MH after vitrectomy occurs rarely, with an incidence of 0.60% in our 11-year retrospective review. While both tangential and anteroposterior traction are thought to be central to the pathogenesis of idiopathic MH, subsequent studies using OCT have demonstrated that a predominantly anteroposterior orientation of tractional forces play a more crucial role^13^. Postoperative MH in the setting of absent vitreous, and therefore, no anteroposterior traction, however, raise questions regarding the responsible pathophysiology and underlying mechanisms^3,11^. In the present study, we examined our own experiences with this surgical complication, describing both the clinical spectrum and treatment outcomes, and have identified possible factors predictive of long-term prognosis.

There are currently two main theoretical mechanisms proposed for the aetiology of a secondary MH formation: the tangential forces from the formation of an ERM and the development of CME^3^. However, in our series, we did not detect any cases of CME after the primary vitrectomy and prior to the diagnosis of secondary MH. Instead, we noted a greater prevalence of ERM, as detected on the OCT images of 27 distinct eyes (71%) overall, both before and after the initial primary vitrectomy. These findings are similar to those of a recent study by Khurana *et al*.^14^, in which no eyes from 14 cases were found to have postoperative CME, but all eyes (100%) had ERM (Table 5). It is important to note that in our series, OCT imaging was not available during the immediate post-operative period and prior to secondary MH diagnosis for all the cases, either due to the difficulty in capturing OCT images, such as after pneumatic tamponade, or irregular follow-up visits from the patients. Additionally, fluorescein angiography was not regularly performed after vitrectomy. Therefore, it is difficult to conclusively state that CME was not a factor for secondary MH development. However, it is revealing that we additionally observed secondary MH formation even in eyes that had received intraoperative Ozurdex®, which is a sustained-release intravitreal dexamethasone implant that, in theory, should drastically reduce ocular inflammation and CME.

**Table 5:** Literature review — secondary macular hole formation after vitrectomy in different studies.

Authors, Journal (Year)	Secondary MH after PPV, Eyes (Incidence)	Mean Age, Years ± SD (Range)	Most Common Diagnosis, Eyes (%)	Mean Interval Between Primary PPV and MH Diagnosis, Month (Range)	ERM/CME Detected Prior to MH Diagnosis, Eyes (%)	High Myopia, Eyes (%)	MH Surgical Outcomes: Successful Closure Rate/Rate of Multiple Operations	Final Visual Outcome, Snellen’s VA (range): Change in VA	Study Conclusions and Key Findings
Lee *et al*.^2^, *Retina (2010)*	10 (0.2% over 5.3 years)	56 ± 16 (27–78)	RD 5 (50%); VH due to PDR 4 (40%); Idio-pathic ERM 1 (10%)	26 (0.6–168)	ERM 4 (40%)/CME 1 (10%)	3 (30%)	9 (90%) closed/0 multiple ops	20/460 (20/21 to counting fingers): 2-line decrease, 2 eyes (20%)	Relatively favourable outcomes, final VA dependent on underlying ocular pathology.
Garcia-Arumi *et al*.^18^, *Retina (2011)*	14 (0.6% after RD repair over 13 years)	52 (29–70)	Macula-off RD 14 (100%)	11 (0.8–78)	ERM 45% overall/CME no data	3 (21%)	14 (100%) closed/0 multiple ops	20/100 (20/28–20/400): 14 (100%) improved compared to pre-op	Favourable MH closure rate, though limited functional VA outcome.
Schlenker *et al*.^3^, *Can J Ophthalmol (2012)*	9 (0.9% after RD repair over 4.5 years)	49 (9–73)	RD 9 (100%): macula-off 8 (89%), re-current 5 (56%)	median 4 (0.07–22)	ERM 2 (22%)/CME no data	1 (11%)	8 (89%) closed/2 (22%) multiple ops	20/200 (20/50 to counting fingers): 1-line increase, 5 eyes (56%)	CME may play a prominent role. Favourable MH closure rates, though visual prognosis remains guarded.
Fabian *et al*.^8^, *Retina (2012)*	7 (1.1% after RD repair over 4.5 years)	60 ± 10 (50–79)	RD 7 (100%): macula-off 3 (30%)	20 (1–48)	ERM 2 (29%)/CME none	(no data)	5 out of 7 (71%) closed/1 (14%) multiple ops	20/74 (20/25–20/480): 3 (43%) improved compared to pre-op	Suggests iatrogenic trauma during PPV, vitreoschisis, ILM traction may cause secondary MH.
Gao *et al*.^10^, *Am J Ophthalmol (2013)*	8 (19% after foveoschisis repair over 10 years)	69 ± 13 (51–83)	Myopic foveoschisis (100%)	1.6 (1–3)	(no data)	Myopia >6D or AXL >26.5 mm: 8 (100%)	(no data)	(no data)	Chorioretinal atrophy/posterior staphyloma not significant. Loss of IS/OS line integrity risk factor for secondary MH development.
Medina *et al*.^11^, *Retina (2017)*	15 (no data on incidence)	64 (50–86)	RD 15 (100%): macula-off 9 (60%), re-current 9 (60%)	4 (1–13)	ERM 11 (73%)/CME none	Myopia >6D or AXL >26.5 mm: 5 out of 9 (56%)	11 (73%) closed/7 (47%) multiple ops	20/267 (20/60 to hand motions): 2-line increase, 8 eyes (53%)	Secondary MH may be associated with ERM, macula-off RD, recurrent RD, and high myopia. Limited visual improvement even after MH closure.
Khurana *et al*.^14^, *Retina (2017)*	14 (no data on incidence)	61 (43–71)	RD 14 (100%): macula-off RD 6 (43%)	median 15 (1–78)	ERM 14 (100%)/CME none	(no data)	12 out of 12 (100%) closed/0 multiple ops	20/25 (20/20 to counting fingers): 13 (93%) improved compared to pre-op	ERM may play a role in the pathogenesis of MH.
Kang *et al*., *Present Study*	38 (0.6% over 10 years)	57 ± 15 (18–77)	RD 9 (23.7%) with 5 macula-off; secondary ERM 6 (15.8%)	median 2.3 (0.04–91)	ERM 19 (50%)/CME none	AXL >28 mm: 6 (15.8%)	34 out of 36 (94%)/10 (28%) multiple ops	20/86 (20/25 to counting fingers): 3-line increase, 18 (47%)	Occurs most commonly after RD repair, associated with ERM. AXL ≥28 mm and poor BCVA at 3 months associated with limited outcome. History of macula-off RD risk factor for multiple surgeries for MH closure.

AXL = axial length; BCVA = best-corrected visual acuity; CME = cystoid macular oedema; ERM = epiretinal membrane; ILM = internal limiting membrane; IS/OS = inner segment/outer segment; MH = macular hole; RD = retinal detachment; PDR = proliferative diabetic retinopathy; PPV = pars plana vitrectomy; SD = standard deviation; VA = visual acuity; VH = vitreous haemorrhage.

Previous clinical observations have revealed that the presence of ERM may be involved in the late reopening of successfully treated MH^15,16^, suggesting that tractional forces from the ERM could be responsible. However, the risk of ERM formation can be reduced with ILM peeling manoeuvres to remove all residual cortical vitreous^17^. In our series, we noted that ILM peeling had been performed in 20 eyes during the initial primary vitrectomy and ERM recurrence was noted in 9 cases, which may have been because of incomplete ILM removal or an insufficiently broad area of peeling. However, there were several eyes in our series that did not develop postoperative ERM, and simply detecting the presence of a postoperative ERM may not prove causation, instead suggesting heterogeneity in the aetiology of this postoperative complication.

Another possible mechanism responsible for secondary MH may involve the iatrogenic traction created during the induction of posterior vitreous detachment, ERM removal, or ILM peeling manoeuvres. A recent study by Sawaguchi *et al*.^9^ described a fascinating case of an MH developing intraoperatively during vitrectomy for VMTS, imaged on intraoperative OCT. As a result, the authors performed ILM peeling and pneumatic tamponade. Additionally, previous studies consistently show a trend of MH formation after repair of RD involving the macula, but the underlying cause is yet to be fully understood^11,18^. This is similar to our own experience, where the majority of cases were associated with RD repair and multivariate analysis revealed that macula-off RD was the most significant factor contributing to the need for multiple operations for MH closure. Altogether, these findings illustrate the complexity and possibly multifactorial nature of secondary MH formation. In the future, widespread use of intraoperative OCT may enable surgeons to consider the minute real-time changes in retinal architecture and further reduce complications.

Regarding the treatment outcomes, we found that the mean BCVA was improved at the final follow-up and more than 24 eyes (63%) showed greater than 1-line visual gain. This is similar to the results of recent studies by Lee *et al*.^2^ and Khurana *et al*.^14^ which also found good visual recovery after surgery, with a high closure rate. We noted that poor long-term visual prognosis was most significantly associated with very long eyes (axial length ≥28 mm), BCVA at onset, and poor BCVA at postoperative month 3. Association of secondary MH with high myopia has also been found in previous studies^11^, and poorer visual recovery in longer eyes may be explained by the observation that in highly myopic eyes, the macula tends to degenerate and the fovea tends to thin^19^.

In our case series, we observed that multiple operations were required in 10 cases (28%), which is low compared with the results of a study by Medina *et al*.^11^ (7 out of 15 eyes, 47%). However, this may be explained by the diversity of the primary diseases in our series, as the aforementioned study only included eyes with secondary MH after RD repair. In cases requiring multiple operations, we used a maximal approach with a combination of adjuvant surgical manoeuvres such as ILM transplantation and autologous platelet injection for physiologic material to close the MH, longer and more substantial mechanical pressure with injection of silicone oil and heavy silicone oil, and relieving of traction by thorough ILM peeling. Utilizing such methods, we successfully achieved MH closure in 34 eyes (94%). Our findings suggest that, to improve the success rate of a single operation, greater caution may be required for eyes with a larger MH size, presence of perioperative ERM, and history of macula-off RD, although only the last clinical factor was significant in multivariate analysis.

Although, to our knowledge, the current series is the largest describing secondary MH formation, it has several limitations due to its retrospective design with variable follow-up and without appropriate controls. The study population was limited to Asian patients in a tertiary university hospital setting. Finally, irregular patient follow-up and lack of immediate post-operative OCT may have led to delays in MH diagnosis. However, the strengths of this study include a large number of patients with a mean follow-up duration longer than 51 months, and treatment of all the patients at tertiary referral-based centres by experienced surgeons.

In summary, we found that secondary MH occurs rarely after vitrectomy, and the most commonly associated diagnosis was rhegmatogenous RD. Secondary ERM were noted in majority of the cases, suggesting that tangential forces may be the most important cause of this surgical complication. Although we observed that secondary MH can be successfully treated in most cases, patients with a history of macula-off RD may require multiple surgeries for hole closure, and those with very long eyes and poor BCVA at 3 months post-operation may have limited visual prognosis.

## Methods

This retrospective study was conducted at two tertiary referral-based hospitals, Severance Hospital and Gangnam Severance Hospital, affiliated with Yonsei University College of Medicine. Consecutive patients who underwent vitrectomy from January 2006 to December 2016 were examined, and cases that developed a postoperative secondary MH were identified for inclusion. Institutional Review Board (Severance Hospital & Gangnam Severance Hospital, Yonsei University Health System) approval was obtained, and informed patient consent was acquired. The study adhered to the tenets of the Declaration of Helsinki.

We reviewed the complete electronic medical records and multimodal imaging data of the identified cases. Multimodal imaging data, when available, included data from fundus photography, ultra-wide field scanning laser ophthalmoscopy (Optomap®; Optos PLC, Dunfermline, UK), fluorescein and indocyanine-green angiography using a confocal scanning system (HRA-2; Heidelberg Engineering Inc., Heidelberg, Germany), and optical coherence tomography (OCT) (Heidelberg Spectralis from Heidelberg Engineering Inc., Heidelberg, Germany; or Cirrus HD-OCT from Carl Zeiss Meditec, Inc., Dublin, CA, USA).

Basic demographic parameters, including age, sex, follow-up duration, and medical histories, were obtained. The primary diagnosis for vitrectomy prior to secondary MH formation was determined through examination of both electronic charts and imaging data. Patients who had undergone initial surgery for MH were excluded. Macular holes were defined as full-thickness defects of the neurosensory retina at the foveal centre as detected on OCT.

Additional macular pathologies (such as the presence of ERM, retinal/submacular haemorrhages, age-related macular degeneration, retino/foveoschisis, foveal cystic changes, etc.) and vitreoretinal pathologies (proliferative diabetic retinopathies, retinal artery/vein occlusions, VMTS, posterior vitreous detachment, vitreous haemorrhage, etc.) both before and after the primary vitrectomy, as well as the history of surgical interventions (scleral encircling/buckling, intravitreal injections, pneumatic retinopexy, laser photocoagulation, cataract surgery, etc.) were documented.

All vitrectomies were performed using either a 23- or 25-gauge system (Constellation® Vision System; Alcon Laboratories, Fort Worth, TX, USA) depending on the surgeon’s preference. Adjuvant techniques that were utilized during either the primary vitrectomy or subsequent vitrectomies for MH closure were noted, such as ILM peeling and/or transplantation, laser photocoagulation, pneumatic tamponade, silicone oil injection, intravitreal injections, and autologous platelet injection. The number of operations prior to secondary MH formation and the number of subsequently required surgeries for MH closure were noted; MH was considered successfully closed when continuous inner retinal layers without intraretinal cysts were observed on OCT.

The best-corrected visual acuity (BCVA) was recorded by Snellen visual acuity and converted to logarithm of minimal angle of resolution (logMAR) scale for evaluation. We noted all available BCVA data at the following time intervals: prior to primary vitrectomy, onset of MH, and post-MH surgery at postoperative months 1, 3, 6, 12, and 24, and finally, at the most recent follow-up visit.

### Main outcome measures

The primary outcomes were the clinical characteristics associated with secondary MH, which included the indications for initial vitrectomy, features on optical coherence tomography, and adjuvant surgical techniques during the initial surgery. Patients were also grouped according to a 1-line or 3-lines visual gain and a 3-lines visual loss for comparison. Furthermore, in cases of eyes operated on for MH closure, we grouped the patients according to whether a single operation or multiple reoperations for MH closure were required. Thus, secondary outcomes included the change in BCVA at 24 months post-operation for MH closure, clinical factors associated with the need for multiple operations for MH closure and prognostic factors associated with the visual outcomes.

### Statistical analysis

We performed statistical analyses using the IBM SPSS version 22.0 software (IBM Corp., Armonk, NY, USA). Kolmogorov-Smirnov tests were used to analyse the distribution of samples. The independent *t* and chi-square tests were used to compare the groups. Logistic regression analyses were performed to assess the impacts of various patient and treatment factors on the outcomes. All data are presented as mean ± standard deviation. *P* values < 0.05 were considered statistically significant.

## Data Availability

The datasets generated during and/or analysed during the current study are available from the corresponding author on reasonable request.
